# Effect of co‐medications and potential risk factors of high‐dose methotrexate‐mediated acute hepatotoxicity in patients with osteosarcoma

**DOI:** 10.1002/cam4.5936

**Published:** 2023-04-16

**Authors:** Sheng‐Fan Wang, Kuan‐Wei Huang, Yueh‐Ching Chou, Hsin‐Chen Lee, Po‐Kuei Wu, Wei‐Ming Chen, Giun‐Yi Hung, Yuh‐Lih Chang

**Affiliations:** ^1^ Department of Pharmacy Taipei Veterans General Hospital Taipei Taiwan; ^2^ Department and Institute of Pharmacology College of Medicine, National Yang Ming Chiao Tung University Taipei Taiwan; ^3^ Department of Clinical Pharmacy, School of Pharmacy Taipei Medical University Taipei Taiwan; ^4^ Department of Pharmacy, School of Pharmaceutical Sciences National Yang Ming Chiao Tung University Taipei Taiwan; ^5^ Department of Orthopedics and Joint Reconstruction Taipei Veterans General Hospital Taipei Taiwan; ^6^ Therapeutical and Research Center of Musculoskeletal Tumor Taipei Veterans General Hospital Taipei Taiwan; ^7^ Institute of Clinical Medicine, School of Medicine National Yang Ming Chiao Tung University Taipei Taiwan; ^8^ Orthopedic Department of Medicine National Yang Ming Chiao Tung University Taipei Taiwan; ^9^ Division of Pediatric Hematology and Oncology, Department of Pediatrics Taipei Veterans General Hospital Taipei Taiwan; ^10^ School of Medicine College of Medicine, National Yang Ming Chiao Tung University Taipei Taiwan

**Keywords:** hepatotoxicity, high‐dose methotrexate, non‐steroidal anti‐inflammatory drugs, osteosarcoma, proton pump inhibitors, trimethoprim‐sulfamethoxazole

## Abstract

**Background:**

Taiwanese patients frequently experience severe hepatotoxicity associated with high‐dose methotrexate (HD‐MTX) treatment, which interferes with subsequent treatment. Drug–drug interactions occur when MTX is used in combination with proton pump inhibitors (PPIs), trimethoprim‐sulfamethoxazole (TMP‐SMX), or non‐steroidal anti‐inflammatory drugs (NSAIDs). In East Asia, real‐world analyses on the effects of co‐medication and other potential risk factors on the clinical course of HD‐MTX‐mediated acute hepatotoxicity in patients with osteogenic sarcoma (OGS) are limited.

**Methods:**

This cohort study included patients with newly diagnosed OGS who were treated with HD‐MTX between 2009 and 2017 at Taipei Veterans General Hospital. We collected data on the clinical course of HD‐MTX‐mediated acute hepatotoxicity, co‐medications, and other potential risk factors, and analyzed the effects of these factors on the clinical course of HD‐MTX‐mediated acute hepatotoxicity.

**Results:**

Almost all patients with OGS treated with HD‐MTX developed acute hepatotoxicity with elevated alanine aminotransferase (ALT) levels. Most patients with Grade 3–4 ALT elevation failed to recover to Grade 2 within 7 days. Women and children are high‐risk subgroups for HD‐MTX‐mediated elevation of ALT levels. Age is a factor that contributes to the pharmacokinetic differences of HD‐MTX. However, the concurrent use of PPIs, TMP‐SMX, or NSAIDs did not affect the elimination of MTX when administered with adequate supportive therapy.

**Conclusions:**

Co‐administration of PPIs, TMP‐SMX, or NSAIDs may have limited effects on acute hepatotoxicity in well‐monitored and adequately pre‐medicated patients with OGS undergoing chemotherapy with HD‐MTX. Clinicians should pay particular attention to ALT levels when prescribing HD‐MTX to children and women.

## 1 INTRODUCTION

Osteogenic sarcoma (OGS) is the most common type of primary bone cancer.^1^ Approximately 60 newly diagnosed cases of OGS have been reported in Taiwan annually,^2^ its peaks in the second decade of life, and accounting for 2% of all childhood cancers.^3^ OGS is sometimes mistaken for growth pain, resulting in delayed diagnosis and treatment.^4^, ^5^ Previously, amputation was considered an essential treatment for OGS. Currently, a combination of treatment modalities, including neoadjuvant and adjuvant chemotherapy and limb‐sparing tumor excisional surgery, has become the standard of care for OGS.^6^ Moreover, patients with OGS and a higher rate of tumor necrosis after neoadjuvant chemotherapy have a better prognosis than those with a lower rate, and tumor necrosis has become an important independent prognostic factor.^7^, ^8^

According to the latest National Comprehensive Cancer Network Guidelines (Bone Cancer, version 2.2023), high‐dose methotrexate (HD‐MTX), adriamycin, and cisplatin (the MAP regimen) are the recommended first‐line chemotherapies according to the results of the EURAMOS‐1 trial.^9^ HD‐MTX is the primary agent in the MAP regimen that causes acute severe hepatotoxicity.^10^ Since the prevalence of hepatitis B and C is significantly higher in East Asia compared with that in Western countries,^11^, ^12^ the MAP regimen used in the EURAMOS‐1 trial containing up to 12 doses of HD‐MTX that may cause severe hepatotoxicity and increase the risk of hepatitis B or C reactivation in hepatitis carriers and its use among East Asian patients raises concerns. Therefore, some clinicians in Asian countries tend to reduce the frequency of HD‐MTX use and replace it with high‐dose ifosfamide to avoid repeated acute hepatotoxicity.^13^, ^14^ Most patients with OGS in Taiwan are treated at Taipei Veterans General Hospital (TVGH). Hung et al. demonstrated the results of OGS treatment using TVGH protocols and showed that reducing the frequency of HD‐MTX use and replacing it with high‐dose ifosfamide did not decrease patient survival.^6^, ^8^ However, data on the clinical course of HD‐MTX‐mediated hepatotoxicity have not been analyzed and reported in these studies.

HD‐MTX‐mediated hepatotoxicity is extremely common and can increase serum transaminase levels several‐fold, even when a leucovorin rescue dose is administered. However, elevated bilirubin levels were rare.^15^ A previous study showed that HD‐MTX‐induced hepatotoxicity can last for 10–14 days in patients with primary central nervous system lymphoma^15^ and can delay the timing of sequential chemotherapy. To prevent HD‐MTX toxicity, therapeutic drug monitoring (TDM) and implementation of various prophylactic measures such as hydration, urinary alkalization, and leucovorin rescue are required.^16^, ^17^ For patients with OGS, those receiving up to 12 g/m^2^ of MTX and showing peak concentrations (4 h after MTX administration) of ≥1000 μM have a better prognosis.^18^ At this exceptionally high dose, the MTX concentrations should be monitored at 4, 24, 48, and 72 h after administration to observe the elimination pattern, and the dose of leucovorin must be adjusted according to the MTX concentration, which must generally be administered continuously every 6 h until the MTX concentration decreases to <0.05–0.1 μM.^16^

Patients with OGS often require medication to treat the disease or the side effects of chemotherapy. However, drug–drug interactions (DDIs) may occur when MTX is used in combination with other medications, such as proton pump inhibitors (PPIs), trimethoprim‐sulfamethoxazole (TMP‐SMX) for *Pneumocystis jirovecii* pneumonia prophylaxis, and non‐steroidal anti‐inflammatory drugs (NSAIDs) for pain control. PPIs act as chemosensitizers by reversing the acidic environment of tumors.^19^ However, PPIs may lead to an increased incidence of MTX toxicity due to delayed elimination of MTX/MTX metabolites.^20^ In addition, both TMP‐SMX and NSAIDs potentially lead to an increases risk of MTX toxicity.^21^, ^22^ Taken together, DDIs between MTX and its co‐medications are frequently encountered and are of great concern when treating OGS.

To further improve the strategies for preventing HD‐MTX toxicity among Asian patients and provide real‐world evidence, this retrospective cohort study aimed to investigate the incidence of acute hepatotoxicity in Taiwanese patients treated with HD‐MTX, the onset and recovery time of hepatotoxicity with appropriate premedication and leucovorin rescue, and the effect of co‐medications and potential risk factors of HD‐MTX‐mediated hepatotoxicity.

## 2 MATERIALS AND METHODS

### 2.1 Study design

This retrospective cohort study was approved by the Institutional Review Board (IRB) of TVGH, Taiwan (IRB‐TPEVGH no. 2019‐08‐007BC). This study was divided into three parts. Part 1 involves the determination of the incidence, timing, and duration of HD‐MTX‐mediated acute hepatotoxicity; Part 2 involves the evaluation of the potential risk factors of HD‐MTX‐mediated acute hepatotoxicity; and Part 3 involves the assessment of the effects of DDIs between HD‐MTX and co‐medications on acute hepatotoxicity and survival outcomes of OGS patients.

### 2.2 Data sources and research tools

Data were collected from the electronic medical record system of TVGH. The extracted variables are listed in Table S1. The research variables and operational definitions are described in the Supplementary Methods. Patients were included from January 1, 2009, to December 31, 2017. Patients who (1) were newly diagnosed with OGS, (2) were treated with HD‐MTX, and (3) underwent adequate preventive measures were included (Table S2). In contrast, patients (1) with a history of liver disease (2) with a personal history of alcoholism, (3) whose treatment did not follow the TVGH OGS 2008 or M2 protocol, (4) who received chemotherapy at other medical institutions, and (5) whose diagnoses were changed or unspecified were excluded. The additional exclusion criteria for Part 3 were as follows: (1) patients with protocol violations, such as severe disease that could not be controlled by scheduled chemotherapy or organ insufficiency, and (2) patients with existing risk factors, such as disseminated disease. Figure S1 shows the data processing and patient numbers.

### 2.3 Evaluation and measurement of research results

The fifth edition of the Common Terminology Criteria for Adverse Events (CTCAE 5.0) was used as the determined criteria to evaluate the grade of MTX side effects, including alanine aminotransferase (ALT), aspartate aminotransferase (AST), alkaline phosphatase (ALP), bilirubin, and creatinine elevations, white blood cell (WBC) count decrease, platelet (PLT) count decrease, and anemia. Blood concentrations of MTX at 4 (peak), 24, and 72 h were evaluated to measure MTX pharmacokinetics. The time of the first disease progression was regarded as the observation endpoint to assess progression‐free survival (PFS). Observations continued until disease progression, loss to follow‐up, death, or the end of the study period (February 1, 2019).

### 2.4 Statistical methods

Detailed statistical analyses are described in the Supplementary Methods. SAS version 9.4 was used for the raw data processing. STATA software version 15 and GraphPad Prism were used to determine the correlation between co‐medication or delayed chemotherapy and survival. *p* ≤ 0.05 was considered significant. Scientific Data Analysis and Graphing Software version 12.5 were used to draw a chart of ALT level trends after HD‐MTX administration.

## 3 RESULTS

### 3.1 Patients' characteristics

A total of 117 eligible patients with OGS were included in this study. The majority of the patients were male (58.12%), and all were of Han Chinese ethnicity. None of the patients were hepatitis B or C carriers. Most of the patients were adolescents (47.86%), had localized disease over the extremities (94.02%), had non‐metastatic status (79.49%), and had conventional histological cell types (95.40%). All the patients had well‐preserved renal function. Additionally, 71.43% were good responders (tumor necrosis rate ≥ 90%) to neoadjuvant chemotherapy. A total of four courses of HD‐MTX were scheduled according to our OGS chemotherapy protocol; however, most (79.49%) of the patients received 1–4 courses of MTX in this study. The initial dosage of MTX was 12 g/m^2^, which was escalated to 13 g/m^2^ if the 4 h peak concentration was lower than 1000 μM. In addition, the dosage of MTX was tapered if significant adverse effects, such as an increase in bilirubin or creatinine, still occurred, even with adequate hydration, alkalization, and leucovorin rescue, and there were no other obvious contributing factors (e.g., comedication or underlying liver diseases). The mean HD‐MTX dose in our study population was 11.85 ± 0.60 g/m^2^. Twenty‐seven patients (23%) were given adjusted doses of HD‐MTX (increased dosage: 15 patients; decreased dosage: 12 patients). The baseline characteristics of the study population are shown in Table S3.

### 3.2 Adverse effects caused by high‐dose methotrexate

The clinical variates in total 117 patients with 485 courses of MTX were collected. Not all courses of MTX had sufficient clinical data to be analyzed. First, 117 patients who received a total of 476 courses of MTX were evaluated for ALT levels. Grade 3–4 ALT elevations were seen in 104 of the 117 patients (88.89%), while 299 of the 476 courses of MTX (62.82%) resulted in a Grade 3–4 ALT elevation. The association with the hepatitis carrier status of the patient was limited because none of the patients were hepatitis B or C carriers. All patients with Grade 3–4 increases in ALT levels recovered and no long‐term complications of liver toxicity occurred. The duration of the course of MTX from the beginning of MTX to the development of Grade 3–4 ALT increase (mean ± SD) was 1.36 ± 0.66. Most Grade 3–4 ALT increases occurred during the first course of MTX (64.1%).

Regarding AST levels, 111 patients who received a total of 332 MTX treatments were evaluated. Twenty‐nine of the 111 patients (26.12%) experienced a Grade 3–4 increase in AST, while 34 of the 332 courses of MTX (10.24%) resulted in a Grade 3–4 increase in AST. None of the patients experienced Grade 3–4 ALP or creatinine increases. The patients who were administered 135 courses (27.95%), 36 courses (7.63%), and 53 courses (10.93%) of MTX developed Grade 3–4 decrease in leukocyte count, decrease in PLT count, and anemia, respectively. The HD‐MTX‐mediated increase in ALT levels is an obvious adverse effect that requires further investigation. The incidences of adverse effects after HD‐MTX administration are shown in Table 1.

**TABLE 1:** Incidence of HD‐MTX‐induced adverse effects.

Total patients (N = 117, 485 courses)	None toxicity	CTCAE^a^ Grade 1	CTCAE Grade 2	CTCAE Grade 3	CTCAE Grade 4
ALT increase (*N* = 117, 476 courses)					
Courses (%)	14 (2.94%)	82 (17.23%)	81 (17.02%)	189 (39.71%)	110 (23.11%)
Patients^b^ (%)	0	2 (1.71%)	11 (9.40%)	57 (48.72%)	47 (40.17%)
AST increase (*N* = 111, 332 courses)					
Courses (%)	182 (54.82%)	102 (30.72%)	14 (4.22%)	31 (9.34%)	3 (0.90%)
Patients^b^ (%)	28 (25.23%)	45 (40.54%)	9 (8.11%)	26 (23.42%)	3 (2.70%)
ALP increase (*N* = 113, 320 courses)					
Courses (%)	294 (91.88%)	26 (8.13%)	0	0	0
Patients^b^ (%)	90 (79.65%)	23 (20.35%)	0	0	0
Creatinine increase (N = 104, 295 courses)					
Courses (%)	292 (98.98%)	3 (1.02%)	0	0	0
Patients^b^ (%)	101 (97.12%)	3 (2.88%)	0	0	0
White blood cell decrease (*N* = 117, 483 courses)					
Courses (%)	151 (31.26%)	77 (15.94%)	120 (24.84%)	100 (20.70%)	35 (7.25%)
Patients^b^	5 (4.27%)	10 (8.55%)	29 (24.79%)	47 (40.17%)	26 (22.22%)
Platelet decrease (*N* = 117, 472 courses)					
Courses (%)	328 (69.49%)	96 (20.34%)	12 (2.54%)	10 (2.12%)	26 (5.51%)
Patients^b^ (%)	48 (41.03%)	36 (30.77%)	7 (5.98%)	6 (5.13%)	20 (17.09%)
Anemia (*N* = 117, 485 courses)					
Courses (%)	159 (32.78%)	72 (14.85%)	201 (41.44%)	53 (10.93%)	0
Patients^b^ (%)	10 (8.55%)	9 (7.69%)	59 (50.43%)	39 (33.33%)	0

*Note*: The information on bilirubin levels is insufficient.

Abbreviations: ALP, alkaline phosphatase; ALT, alanine transaminase; AST, aspartate transaminase.

^a^ Common Terminology Criteria Adverse Events v 5.0.

^b^ Patients experienced the most serious level of adverse effect.

To further clarify hepatotoxicity, we divided the patients into two groups: those received less than or equal to the median course of MTX (≤4) and those received greater than the median course of MTX (>4), and compared the differences in the incidence of ALT and AST elevation between these two groups. Of the 91 patients treated with ≤4 courses of MTX, 83 patients (91.21%) experienced a Grade 3–4 increase in ALT, while 21 (80.77%) of the 26 patients treated with >4 courses of MTX experienced a Grade 3–4 increase in ALT (Table S4). Similarly, 24 of the 86 patients (27.91%) who received ≤4 courses of MTX experienced a Grade 3–4 increase in AST, whereas 5 of the 25 patients (20%) who received >4 courses of MTX experienced a Grade 3–4 increase in AST (Table S4). Our results suggest that receiving more than four courses of MTX does not increase the incidence of Grade 3–4 ALT and AST elevations.

According to the treatment guidelines, the next chemotherapy session was scheduled on the seventh day after HD‐MTX therapy. Generally, to ensure safety, sequential chemotherapy is not administered until the severity of adverse effects is reduced to Grade 2 or below. A total of 299 records (≥Grade 3 increase in ALT levels) were analyzed. The mean baseline ALT level was 28.04 ± 1.04 U. The maximum ALT level (688.90 ± 33.09 U) was seen within 24 h after MTX administration. Most importantly, the ALT level remained high on the seventh day after MTX administration (340.19 ± 24.35 U). The severity of increase in ALT was subsequently reduced to Grade 2 (202.68 ± 23.90 U) on the 10th day after MTX administration. Figure S2 shows the trends in ALT levels after HD‐MTX treatment.

### 3.3 Risk factors associated with HD‐MTX‐mediated ALT increase and delayed subsequent chemotherapy

Increased ALT levels are the most common cause of HD‐MTX‐mediated acute liver toxicity. After adjustment of age, gender, body mass index, co‐medications (including PPIs, TMP‐SMX, and NSAIDs), MTX dose, and MTX blood concentration within 4/24/72 h after administration, the maximum ALT level decreased in the adolescent and adult subgroups (−217.56 ± 84.27 U; −319.67 ± 98.88 U, Table 2). Additionally, the maximum ALT level was higher in women compared with that in men (+316.35 ± 72.85 U, Table 2). Younger age and female sex are the potential risk factors for HD‐MTX‐mediated acute hepatotoxicity. However, no significant correlation was observed between the maximum ALT level and body mass index (BMI), co‐medications, or TDM of MTX (Table 2).

**TABLE 2:** Multivariate analysis of risk factors associated with maximum ALT level.

	b (SE)	*p*‐Value		b (SE)	*p*‐Value
Age^a^			TMP‐SMX		
Child	Ref		Non‐user	Ref	
Adolescent	−217.56 (84.27)	0.0325	User	82.23 (42.68)	0.0582
Adult	−319.67 (98.88)	0.0120	NSAIDs		
Gender			Non‐user	ref	
Male	Ref		User	−182.73 (91.04)	0.0600
Female	316.35 (72.85)	< 0.0001	MTX dose		
BMI				84.01 (53.67)	0.1184
Normal weight	Ref		MTX 4 h level (μM)		
Underweight	−104.81 (72. 27)	0.1661	< 1000	Ref	
Pre‐obesity	120.56 (77.24)	0.1266	≥ 1000	−79.47 (53.94)	0.1466
Obesity	49.59 (98.47)	0.6174	MTX 24 h level (μM)		
PPIs			< 10	Ref	
Non‐user	Ref		≥ 10	18.94 (76.51)	0.8064
User	−30.06 (54.59)	0.5849	MTX 72 h level (μM)		
			< 0.1	Ref	
			≥ 0.1	−5.13 (50.70)	0.9198

Abbreviations: BMI, body mass index; NSAIDs, nonsteroidal anti‐inflammatory drugs; MTX, methotrexate; PPIs, proton pump inhibitors; TMP‐SMX, trimethoprim‐sulfamethoxazole.

^a^ Male: child 0**–**12 years; adolescent 13**–**17 years; adult 18 years or older. Female: child 0**–**11 years; adolescent 12**–**16 years; adult 17 years or older.

To clinically evaluate the liver toxicity of HD‐MTX, we further evaluated the potential risk factors for delayed chemotherapy after HD‐MTX. Because only a few patients (three patients) delayed the next course of chemotherapy after HD‐MTX for more than 1 week, we defined delaying subsequent chemotherapy for more than 3 days as delayed chemotherapy after HD‐MTX and further analyzed its impact. Subsequent chemotherapy was delayed in 85 of 117 patients (72.65%) after HD‐MTX, while the other 32 patients (27.35%) continued with subsequent chemotherapy without delay. Moreover, younger age (odds ratio [OR] for adolescent: 0.47, 95% confidence interval [CI]: 0.27–0.82; OR for adult: 0.35, 95% CI: 0.19–0.63) and female sex (OR: 3.0, 95% CI: 1.15–7.83) are potential risk factors of HD‐MTX‐mediated delayed subsequent chemotherapy (Table S5). Furthermore, no significant correlations were observed between delayed chemotherapy after HD‐MTX treatment and BMI, co‐medications, or TDM of MTX (Table S5). In contrast, delayed chemotherapy after HD‐MTX treatment did not significantly affect the progression‐free survival of patients with OGS (Figure S3).

### 3.4 Effects of the risk factors associated with HD‐MTX‐mediated acute hepatotoxicity on MTX pharmacokinetics

Monitoring the blood concentration of MTX may be useful for evaluating the efficacy and adverse effects of HD‐MTX. First, we evaluated the risk factors for peak MTX concentration (MTX 4 h ≥ 1000 μM, related to efficacy). After adjustment for multivariate factors, the adult group, group whose MTX dose was increased by 1 g/m^2^, and PPI‐used subgroups had higher probability of experiencing an MTX peak concentration of ≥1000 μM compared with the reference group (adjusted OR for adults: 4.12, 95% CI: 1.53–11.09; adjusted OR for MTX dose: 2.93, 95% CI: 1.58–5.44; adjusted OR for PPIs: 2.22, 95% CI: 1.02–4.8, Table 3). Conversely, the NSAIDs usage subgroup had a lower probability of experiencing peak MTX concentration of ≥1000 μM compared with the reference group (adjusted OR: 0.23, 95% CI: 0.09–0.58, Table 3).

**TABLE 3:** Multivariate analysis of association between HD‐MTX peak level (4 h MTX concentration ≥ 1000 μM) and potential risk factors.

	Odds ratio (95% CI)	*p*‐Value		Odds ratio (95% CI)	*p*‐Value
Age^a^			Baseline creatinine clearance		
Child	1.0 (Ref)			0.99 (0.98–1.01)	0.2693
Adolescent	0.91 (0.45–1.83)	0.7953	MTX dose		
Adult	4.12 (1.53–11.09)	0.0051		2.93 (1.58–5.44)	0.0006
Gender			PPIs		
Male	1.0 (Ref)		Non‐user	1.0 (Ref)	
Female	0.56 (0.31–1.03)	0.0622	User	2.22 (1.02–4.80)	0.0433
BMI			TMP‐SMX		
Normal weight	1.0 (Ref)		Non‐user	1.0 (Ref)	
Underweight	1.27 (0.57–2.81)	0.5528	User	0.91 (0.48–1.74)	0.7830
Pre‐obesity	1.40 (0.48–4.09)	0.5364	NSAIDs		
Obesity	0.58 (0.23–1.45)	0.2435	Non‐user	1.0 (Ref)	
			User	0.23 (0.09–0.58)	0.0016

Abbreviations: BMI, body mass index; HD‐MTX, high‐dose methotrexate; NSAIDs, nonsteroidal anti‐inflammatory drugs; PPIs, proton pump inhibitors; TMP‐SMX, trimethoprim‐sulfamethoxazole.

^a^ Male: child 0–12 years; adolescent 13–17 years; adult 18 years or older. Female: child 0–11 years; adolescent 12–16 years; adult 17 years or older.

The concentration of MTX is expected to reduce to <10 μM and <0.1 μM at 24 and 72 h after administration, respectively.^16^, ^23^, ^24^ Patients treated with 38 (8.84%) and 94 courses (21.81%) of MTX experienced delayed MTX elimination at 24 and 72 h after administration, respectively. Increased age might contribute to delayed MTX elimination (adjusted OR for 24‐h MTX concentration of ≥10 μM: 1.10, 95% CI: 1.03–1.17; adjusted OR for 72‐h MTX concentration of ≥0.1 μM in the adult subgroup: 7.61, 95% CI: 2.98–19.4, Tables 4 and 5). Other potential risk factors such as sex, BMI, and co‐medications might not interfere with the MTX elimination; moreover, the baseline creatinine clearance has a little impact on 72‐h MTX elimination (adjusted OR: 0.97, 95% CI: 0.96–0.99, Table 5). The effects of creatinine clearance on MTX pharmacokinetics in patients with well‐preserved renal function are limited (Tables 3, 4, 5).

**TABLE 4:** Multivariate analysis of the association between delayed elimination of HD‐MTX (24‐h MTX concentration ≥ 10 μM) and potential risk factors.

	Odds ratio (95% CI)	*p*‐Value		Odds ratio (95% CI)	*p*‐Value
Age^a^			MTX dose		
	1.10 (1.03–1.17)	0.0060		1.34 (0.57–3.12)	0.5048
Gender			PPIs		
Male	1.0 (Ref)		Non‐user	1.0 (Ref)	
Female	0.90 (0.32–2.49)	0.8357	User	1.04 (0.37–2.92)	0.9365
BMI			TMP‐SMX		
Normal weight	1.0 (Ref)		Non‐user	1.0 (Ref)	
Underweight	1.34 (0.41–4.40)	0.6310	User	1.46 (0.62–3.43)	0.3818
Pre‐obesity	0.43 (0.10–1.82)	0.2540	NSAIDs		
Obesity	0.94 (0.23–3.82)	0.9353	Non‐user	1.0 (Ref)	
Baseline creatinine clearance			User	1.62 (0.36–7.33)	0.5330
	0.97 (0.94–1.00)	0.0594			

Abbreviations: BMI, body mass index; HD‐MTX, high‐dose methotrexate; NSAIDs, nonsteroidal anti‐inflammatory drugs; PPIs, proton pump inhibitors; TMP‐SMX, trimethoprim‐sulfamethoxazole.

^a^ Age was adopted as a continuous variable because of the absence of delayed elimination of 24‐h MTX concentration in the child subgroup.

**TABLE 5:** Multivariate analysis of the association between delayed elimination of HD‐MTX (72‐h MTX level ≥ 0.1 μM) and potential risk factors.

	Odds ratio (95% CI)	*p*‐Value		Odds ratio (95% CI)	*p*‐Value
Age^a^			Baseline creatinine clearance		
Child	1.0 (Ref)			0.97 (0.96–0.99)	0.0020
Adolescent	1.23 (0.45–3.39)	0.6870	MTX dose		
Adult	7.61 (2.98–19.40)	<0.0001		1.38 (0.85–2.24)	0.1930
Gender			PPIs		
Male	1.0 (Ref)		Non‐user	1.0 (Ref)	
Female	0.64 (0.34–1.21)	0.1705	User	0.68 (0.34–1.33)	0.2585
BMI			TMP‐SMX		
Normal weight	1.0 (Ref)		Non‐user	1.0 (Ref)	
Underweight	0.83 (0.33–2.06)	0.6869	User	1.40 (0.83–2.36)	0.2073
Pre‐obesity	0.64 (0.31–1.35)	0.2400	NSAIDs		
Obesity	0.86 (0.36–2.07)	0.7446	Non‐user	1.0 (Ref)	
			User	1.72 (0.64–4.65)	0.2845

Abbreviations: BMI, body mass index; HD‐MTX, high‐dose methotrexate; NSAIDs, nonsteroidal anti‐inflammatory drugs; PPIs, proton pump inhibitors; TMP‐SMX, trimethoprim‐sulfamethoxazole.

^a^ Male: child 0–12 years; adolescent 13–17 years; adult 18 years or older. Female: child 0–11 years; adolescent 12–16 years; adult 17 years or older.

### 3.5 Effects of drug–drug interactions between HD‐MTX and co‐medications on survival outcomes

During the observation period, 12 (26.1%) and 24 patients (37.5%) in the PPI subgroup and non‐PPI subgroups, respectively, experienced disease progression. A total of 22 (31.9%) and 14 patients (34.1%) in the TMP‐SMX subgroup and non‐TMP‐STX‐used groups, respectively, experienced disease progression. A total of 5 (29.3%) and 31 patients (33.3%) in the NSAID subgroup and non‐NSAID subgroups, respectively, experienced disease progression.

**FIGURE 1:**
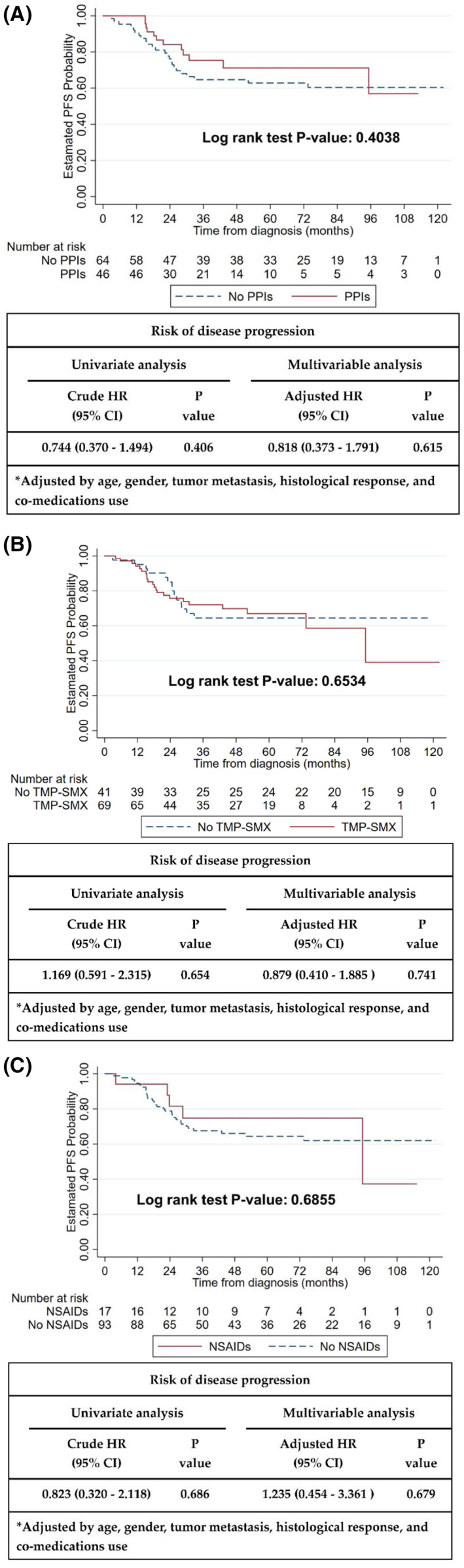
Effects of proton pump inhibitors, TMP‐SMX, and NSAIDs on the risk of disease progression and progression‐free survival. (A) PPIs, proton pump inhibitors; (B) TMP‐SMX, trimethoprim‐sulfamethoxazole; (C) NSAIDs, nonsteroidal anti‐inflammatory drugs; HR, hazard ratio.

Kaplan–Meier survival curve analysis and Cox proportional hazards regression analysis showed that the use of concurrent co‐medications, such as PPIs, TMP‐SMX, and NSAIDs, did not interfere with PFS (PPIs: *p* = 0.4038; TMP‐SMX: *p* = 0.6534; NSAIDs: *p* = 0.6855, Figure 1A–C). PPIs, TMP‐SMX, and NSAIDs did not affect the risk of disease progression after multivariable adjustment (adjusted hazard ratio [HR] for PPIs: 0.818, 95% CI: 0.373–1.791; adjusted HR for TMP‐SMX: 0.879, 95% CI: 0.41–1.885; adjusted HR for NSAIDs: 1.235, 95% CI: 0.454–3.361, Figure 1A–C).

## 4 DISCUSSION

Almost all patients experienced an increase in ALT levels. Most patients developed Grade 3–4 ALT increase after HD‐MTX administration (Table 1). A Grade 3–4 ALT increase was observed in 59% and 34.9% of the patients in single‐center prospective studies from Norway and Hungary, respectively.^25^, ^26^ The incidence of ALT increase was higher in our study than in Western countries. Our results illustrate the clinical course of acute hepatotoxicity in patients with OGS. The ALT level reached a maximum value (689 U) 24 h after HD‐MTX administration (Figure S2). The average ALT level remained high (340 U) on the seventh day after HD‐MTX administration. On the 10th day after HD‐MTX administration, the ALT level reduced to 203 U, which was close to CTCAE Grade 2 (general eligibility criteria for chemotherapy). This finding is consistent with that of a previous study on primary CNS lymphoma.^15^ Therefore, it is necessary to carefully monitor liver function and make appropriate adjustments according to the patient's clinical status.

PPIs may only affect the peak concentration of MTX (Tables 3, 4, 5). Some evidence showed that the PPIs might delay the MTX elimination.^20^, ^27^ However, Reeves et al. demonstrated no significant relationship between the concurrent use of PPIs and the delayed elimination of MTX using a repeated‐measures regression model.^28^ The discrepancy in these results might be attributed to differences in statistical methods (both our study and the Reeves study were adjusted for repeated measurements), premedication status, and ethnic groups. In addition, compensatory metabolic pathways and gene variants may contribute to this difference.^29^, ^30^

Our study revealed that TMP‐SMX and HD‐MTX did not affect the 4‐/24‐/72‐h MTX concentrations (Tables 3, 4, 5). Watts et al. also concluded that TMP‐SMX did not alter MTX pharmacokinetics.^31^ Interestingly, the NSAIDs group has lower risk of experiencing a peak MTX concentration of ≥1000 μM compared with the unused group (OR = 0.23, 95% CI: 0.09–0.58, Table 3). However, treatment with NSAIDs did not increase the PFS (Figure 1C). Thyss et al. revealed that patients who concurrently use ketoprofen and MTX may experience serious side effects or even death.^32^ However, Suzuki et al. concluded that the combined use of MTX and NSAIDs did not significantly delay the elimination of MTX.^27^ Therefore, further large‐scale, prospective, and rigorous studies are warranted to confirm these findings.

This study investigated the potential risk factors that might interfere with MTX pharmacokinetics. Age and MTX dose may have affected the peak MTX concentration (Table 3). Additionally, older age contributed to the delayed elimination of the 24‐/72‐h MTX concentration (Tables 4 and 5). Logically, patients with a higher basal creatinine clearance rate may have a decreased odds ratio for delayed MTX elimination (Table 5). Previous studies have also demonstrated that younger patients have a lower 24‐h MTX concentration, which might be due to the larger distribution volume and shorter half‐life of MTX in this patient group.^33^, ^34^ Moreover, Reeves et al. revealed that the baseline creatinine value in the group with delayed elimination of MTX was higher than that in the group without delayed elimination.^28^ These results are consistent with the findings of this study.

Age and sex might have affected the extent of the ALT level increase after HD‐MTX administration (Table 2). Holmboe et al. indicated that younger age, female sex, lower MTX clearance, and higher area under the curve (AUC)_7‐OH‐MTX_:AUC_total_ were potential risk factors affecting the severity of ALT increase after MTX administration.^26^ Additionally, Hegyi et al. suggested that a higher peak concentration of MTX, a higher AUC, and a lower MTX clearance rate were related to hepatotoxicity.^25^ However, the pharmacokinetics may not affect the severity of acute hepatotoxicity (Table 2). TDM frequency, precise blood MTX concentration, use of different statistical methods, premedication status, leucovorin salvage therapy status, idiosyncratic factors, and pharmacogenomics may have contributed to the inconsistencies in these studies. Moreover, Collins et al. reported that children and women with OGS had better survival rates, with more severe grades of adverse effects, compared to the reference group.^35^ Hence, further large‐scale, prospective, and detailed MTX TDM studies are required.

Although Ferrari et al. pointed out a tendency for increased clinical efficacy of MTX when combined with PPIs,^19^ our results demonstrated that PPIs did not have a significant impact on PFS (Figure 1A). The PPI dosages used in these studies were different. Hence, the exact effect of PPIs on the clinical efficacy of OGS treatment requires further investigation in a large‐scale prospective study.

Precise MTX blood concentrations were not available because of the retrospective nature of the study, which may have limited the accuracy of the pharmacokinetic assay. Total bilirubin, gamma‐glutamyl transpeptidase (GGT), and MTX metabolite levels have not been reported. Moreover, only Taiwanese patients from a single experienced medical center were recruited for this study; hence, extrapolation might be limited. However, an expert single medical center (managing the majority of patients with OGS in Taiwan) that uses uniform treatment guidelines might have relatively low heterogeneity, which confers the advantage of this study.

## 5 CONCLUSIONS

Most patients would experience ALT increase after HD‐MTX administration. Therefore, HD‐MTX‐mediated acute hepatotoxicity is a well‐studied clinical issue in patients with OGS. Concurrent use of PPIs, TMP‐SMX, or NSAIDs with HD‐MTX may not be a serious clinical issue in patients receiving proper premedication and salvage therapy. However, it may be vital to consider the risk of HD‐MTX‐mediated acute hepatotoxicity in children and female subpopulations.

## AUTHOR CONTRIBUTIONS

**Sheng‐Fan Wang:** Conceptualization (equal); data curation (equal); formal analysis (equal); funding acquisition (equal); investigation (equal); project administration (equal); resources (equal); validation (equal); visualization (equal); writing – original draft (equal). **Kuan‐Wei Huang:** Conceptualization (equal); data curation (equal); formal analysis (equal); investigation (equal); project administration (equal); resources (equal); validation (equal); visualization (equal); writing – original draft (equal). **Yueh‐Ching Chou:** Conceptualization (equal); resources (equal); supervision (equal). **Hsin‐Chen Lee:** Resources (equal). **Po‐Kuei Wu:** Resources (equal). **Wei‐Ming Chen:** Resources (equal). **Giun‐Yi Hung:** Conceptualization (equal); data curation (equal); formal analysis (equal); funding acquisition (equal); investigation (equal); resources (equal); supervision (equal); validation (equal); visualization (equal); writing – review and editing (equal). **Yuh‐Lih Chang:** Resources (equal); supervision (equal); writing – review and editing (equal).

## CONFLICT OF INTEREST STATEMENT

The authors report no conflict of interest.

## FUNDING STATEMENT

This study was supported by grants from the National Science and Technology Council (MOST111‐2320‐B‐075‐004 and MOST111‐2313‐B‐075‐001‐MY3), TVGH, Taipei, Taiwan (V112C‐047), and the Melissa Lee Cancer Foundation (MLCF‐V111_A11103 and MLCF‐V111_B11106).

## ETHICS STATEMENT

This retrospective cohort study was reviewed by the Institutional Review Board of TVGH, Taiwan (IRB‐TPEVGH no. 2019–08‐007 BC).

## Supporting information


Data S1:

## Data Availability

Data sharing is not applicable to this article as no new data were created or analyzed in this study.
