# Supplementary material for: Effect of co‐medications and potential risk factors of high‐dose methotrexate‐mediated acute hepatotoxicity in patients with osteosarcoma
Source: Cancer Med. 2023 Apr 16;12(11):12354–64. doi: 10.1002/cam4.5936 (PMC10278458; doi:10.1002/cam4.5936)
Supplement: Supplementary file 1 — Data S1: [file CAM4-12-12354-s001.docx]

**Supplementary methods**

***Research variables and operational definitions***

Patient’s characteristics: (1) gender: male and female; (2) index date: the date when the patient’s electronic medical record cancer information was pathologically diagnosed as osteosarcoma; (3) age: the age at the time of inclusion in the study, in years. Patients were divided into three subgroups (child: male, aged 0–12 years; female, aged 0–11 years) (adolescent: male, aged 13–17 years; female, aged 12–16 years) (adult: male, aged 18 years or older; female, aged 17 years or older) according to a previous study [1]; (4) tumor characteristics: (a) occurrence location: data obtained from the electronic medical record system of the pathology department and divided into three groups: extremities, pelvis, and others. (b) Metastasis: The patients were divided into two groups (yes and no). The original TNM staging of cancer information in the electronic medical record system was used to assess for presence of metastasis. Additionally, the results of computerized tomography of the thoracic system at the time of diagnosis and whole-body bone scan were used to re-confirm the diagnosis; (5) histopathological characteristics: (a) Tumor cell type: data obtained from the electronic medical record system of the pathology department or nuclear magnetic resonance photography report. The osteoblastic, chondroblastic, and fibroblastic cell types were classified as conventional types, and other cell types were classified as non-conventional. (b) Neoadjuvant chemotherapy response rate: data obtained from the electronic medical record system of the pathology department or nuclear magnetic resonance imaging report. A response rate of ≥90% was regarded as a good response, while a response rate of <90% was classified as poor response; (6) number of high-dose methotrexate (HD-MTX) administrations was obtained from the cancer drug treatment record query and medication information of the electronic medical record system (calculation was based on the number of HD-MTX administered by the patient after diagnosis). Continuous variables, such as age, were expressed as mean ± standard deviation. Categorical variables, such as age, sex, tumor characteristics, number of MTX used, and severity of side effects, were expressed as percentages (%).

Outcome variables and definitions: (1) The severity of adverse effects was graded in accordance with the fifth edition of the Common Terminology Criteria for Adverse Events, and the values of the following parameters were obtained from the electronic medical record system: (a) alanine aminotransferase and aspartate aminotransferase: the baseline values before the administration of MTX and all the values ​​after the administration. (b) alkaline phosphatase and creatinine: baseline values before MTX administration and values ​​after administration. (c) white blood count, platelet, and hemoglobin: the baseline values before MTX administration and the lowest value after administration; (2) factors affecting the pharmacokinetics of MTX and hepatotoxicity: (a) body mass index (BMI): calculated as weight in kilograms divided by height in meters squared and classified into underweight, normal, overweight, and obese according to age based on the guidelines of the Health Promotion Administration, Ministry of Health and Welfare, Taiwan. (b) Co-medication usage: obtained from the electronic medical record system and divided into two groups according to whether the indicated co-medication was used in conjunction with MTX during the period of patient’s treatment. (c) MTX dosage: calculated according to the number of grams per unit of body surface area (g/m^2^). (d) The concentration of MTX at the 4 h/24 h/72 h: obtained from the general laboratory report of the electronic medical record system and divided into two groups based on whether the level of MTX: ≥1000 μM (4 h), ≥10 μM (24 h), and ≥0.1 μM (72 h). (e) Renal function: based on the creatinine value before MTX administration obtained through a biochemical test and reported in the electronic medical record system; it was also converted into creatinine clearance and calculated based on the Schwartz and Chronic Kidney Disease Epidemiology Collaboration equations; (3) progression-free survival: the results of computed tomography, magnetic resonance imaging, and whole-body bone scan from the medical record system were evaluated. If it was recorded as progression, metastasis, relapse, death, or occurrence of secondary cancer, it was defined as deterioration of clinical condition, and the time of its occurrence was noted.

***Detailed statistical analyses***

Descriptive statistics were used to present the patient’s characteristics and incidence of side effects (*p* ≤0.05, considered significant). The correlation between the risk factors and hepatotoxicity was investigated using regression analysis. A linear mixed model was used to analyze the correlation between various potential factors and hepatotoxicity (maximum ALT level after HD-MTX, continuous variable). A generalized estimating equation was used to evaluate the relationship between various potential factors and the pharmacokinetic properties of MTX (peak concentration and drug elimination, relevance analysis of categorical variables).

The correlation between co-medication and survival was calculated using STATA software version 15 and the log-rank test; a *p*-value of ≤0.05 was considered significant. Kaplan-Meier (KM) plot analysis was used to estimate the effect of co-medication on PFS. The Cox proportional hazard regression model was used for the univariate and multivariable analyses. Scientific Data Analysis and Graphing Software version 12.5 were used to draw the chart of ALT level trend after HD-MTX administration.

**Reference:**

1. Collins M, Wilhelm M, Conyers R, et al. Benefits and adverse events in younger versus older patients receiving neoadjuvant chemotherapy for osteosarcoma: findings from a meta-analysis. J Clin Oncol. 2013 Jun 20;31(18):2303-12. doi: 10.1200/JCO.2012.43.8598. PubMed PMID: 23669227.

**Supplementary Table 1. Research data and extraction variables**

| Data source | File name | Extraction variable |
| --- | --- | --- |
| Electronic Medical Record System | Hospitalized cases | Patient identification code, name, gender, date of birth |
|  | Patient Information | Drinking status, date of death |
|  | Vital signs | Height, weight, body mass index (BMI), body surface area (BSA) |
|  | Cancer Information | Cancer treatment information (date of diagnosis, cancer stage), cancer drug treatment records (dose, date of administration) |
|  | Admission Note | Admission diagnosis, past medical history, admission record, disease course record |
|  | Discharge Note | Hospitalization, discharge diagnosis |
|  | Biochemical blood test | alanine aminotransferase (ALT), aspartate aminotransferase (AST), alkaline phosphatase, (ALP), total bilirubin (TB), creatinine, blood urea nitrogen (BUN), white blood cell (WBC), platelet count (PLT), hemoglobin (Hgb), DC-band, DC-segment (SEG) |
|  | Therapeutic drug concentration monitoring | MTX dose, 4-, 24-, 48-, 72-, 96-, 120-, 144-, 168-, 192-, 216-, 240-, 264-h after MTX administration in blood concentration |
|  | Medication record | medications, dosage, unit, frequency, route, duration, and pre-medications |
|  | General report | Computed tomography, MRI, whole body bone scan |

**Supplementary Table 2. Adequate preventive measures during the use of high-dose methotrexate**

| Intensity hydration | ≥ 200 mL/m^2^/h for at least 2 h or 100-150 mL/m^2^/h for at least 12 h, and continuing hydration for 24-48 h after administration of MTX; if the patient has a past history of delayed exclusion or side effects, hydration must be longer |
| --- | --- |
| Urine alkalization | Sodium bicarbonate (40-50 meq/L NaHCO_3_ is supplemented before, during and after HD-MTX administration to make urine PH >7) is helpful for avoiding the formation and deposition of MTX crystals in renal tubules, which may cause kidney damage. |
| Leucovorin rescue | 10 doses of leucovorin 10 mg q6h (24 h after the administration of MTX); adjusted leucovorin dosage according to whether the MTX blood concentration monitoring is higher than the MTX upper limit concentration (24h-/48h-/72h- MTX: ≥10/1/0.1 μM) |

**Supplementary Table 3: Patients’ baseline characteristics**

| **Patient’s characteristics** | **Number of patients (N= 117)** |
| --- | --- |
| **Gender** |  |
| **Male** | **68 (58.12%)** |
| **Female** | **49 (41.88%)** |
| **Age^#^** |  |
| **Child** | **28 (23.93%)** |
| **Adolescent** | **56 (47.86%)** |
| **Adult** | **33 (28.21%)** |
| **mean ± SD** | **16.78 ± 7.44** |
| **Tumor** |  |
| **Site** |  |
| **Extremity** | **110 (94.02%)** |
| **Pelvic** | **4 (3.42%)** |
| **Others** | **3 (2.56%)** |
| **Metastasis** |  |
| **Yes** | **24 (20.51%)** |
| **No** | **93 (79.49%)** |
| **Histology** |  |
| **Cell type (N=87)** |  |
| **Conventional*** | **83 (95.40%)** |
| **Non-conventional** | **4 (4.60%)** |
| **Response^†^ (N=98)** |  |
| **Good response** | **70 (71.43%)** |
| **Poor response** | **28 (28.57%)** |
| **Number of MTX course received** | |
| **1–4** | **93 (79.49%)** |
| **5–9** | **22 (18.80%)** |
| **≥10** | **2 (1.71%)** |

^#^ Male: child 0–12 years; adolescent 13–17 years; adult 18 years or older; female: child 0–11 years; adolescent 12–16 years; adult 17 years or older

*Conventional cell types: osteoblastic, chondroblastic, and fibroblastic

†Good response: tumor necrosis rate ≥ 90%; poor response: tumor necrosis rate < 90%

MTX, methotrexate

**Supplementary Table 4. The subgroups analyses for the incidence of HD-MTX-induced ALT/AST increase**

|  | **None toxicity** | **CTCAE***  **grade 1** | **CTCAE**  **grade 2** | **CTCAE**  **grade 3** | **CTCAE**  **grade 4** |
| --- | --- | --- | --- | --- | --- |
| **ALT increase** | | | | | |
| **≦4 courses of MTX (91 patients)** | | | | | |
| **Patients (%)** | **0**  **(0%)** | **1**  **(1.10%)** | **7**  **(7.69%)** | **45**  **(49.45%)** | **38**  **(41.76%)** |
| **>4 courses of MTX (26 patients)** | | | | | |
| **Patients** (%)** | **0**  **(0%)** | **1**  **(3.85%)** | **4**  **(15.38%)** | **12**  **(46.15%)** | **9**  **(34.62%)** |
| **AST increase** | | | | | |
| **≦4 courses of MTX (86 patients)** | | | | | |
| **Patients****  **(%)** | **22 (25.58%)** | **33**  **(38.37%)** | **7**  **(8.14%)** | **22**  **(25.58%)** | **2**  **(2.33%)** |
| **>4 courses of MTX (25 patients)** | | | | | |
| **Patients** (%)** | **6**  **(24.00%)** | **12**  **(48.00%)** | **2**  **(8.00%)** | **4**  **(16.00%)** | **1**  **(4.00%)** |

*Common Terminology Criteria Adverse Events v 5.0

**Patients experienced the most serious level of adverse effect

**Supplementary Table 5. Multivariate analysis of the association between delayed chemotherapy (>3 days) after MTX and potential risk factors**

|  | **Odds ratio**  **(****95% CI)** | ***p*-value** |  | **Odds ratio**  **(95% CI)** | ***p* -value** |
| --- | --- | --- | --- | --- | --- |
| **Age^#^** | | | **TMP-SMX** | | |
| **Child** | **1.0**  **(Ref)** |  | **Non-user** | **1.0**  **(Ref)** |  |
| **Adolescent** | **0.47**  **(0.27-0.82)** | **0.0359** | **User** | **1.20**  **(0.49-2.95)** | **0.6966** |
| **Adult** | **0.35 (0.19-0.63)** | **0.0229** | **NSAIDs** |  | |
| **Gender** | | | **Non-user** | **1.0**  **(Ref)** |  |
| **Male** | **1.0**  **(Ref)** |  | **User** | **0.69**  **(0.23-2.03)** | **0.5020** |
| **Female** | **3.0**  **(1.15-7.83)** | **0.0247** | **MTX dose** | | |
| **BMI** | | |  | **1.55**  **(0.28−3.88)** | **0.2375** |
| **Normal weight** | **1.0**  **(Ref)** |  | **MTX 4 h level (μM)** | | |
| **Underweight** | **0.88**  **(0.40-1.57)** | **0.4135** | **< 1000** | **1.0**  **(Ref)** |  |
| **Pre-obesity** | **0.75**  **(0.27-2.04)** | **0.4588** | **≥ 1000** | **0.86 (0.35-1.93)** | **0.6581** |
| **Obesity** | **0.66**  **(0.40-1.88)** | **0.5872** | **MTX 24 h level (μM)** | | |
| **PPIs** | | | **< 10** | **1.0**  **(Ref)** |  |
| **Non-user** | **1.0**  **(Ref)** |  | **≥ 10** | **0.981**  **(0.82-1.22)** | **0.7412** |
| **User** | **1.74**  **(0.70-4.29)** | **0.2321** | **MTX 72 h level (μM)** | | |
|  | | | **< 0.1** | **1.0**  **(Ref)** |  |
|  | | | **≥ 0.1** | **1.36**  **(0.45-1.87)** | **0.742** |

^#^ Male: child 0**−**12 years; adolescent 13**−**17 years; adult 18 years or older. Female: child 0**−**11 years; adolescent 12**−**16 years; adult 17 years or older. MTX, methotrexate; BMI, body mass index; PPIs, proton pump inhibitors; TMP-SMX, trimethoprim-sulfamethoxazole; NSAIDs, nonsteroidal anti-inflammatory drugs.

**
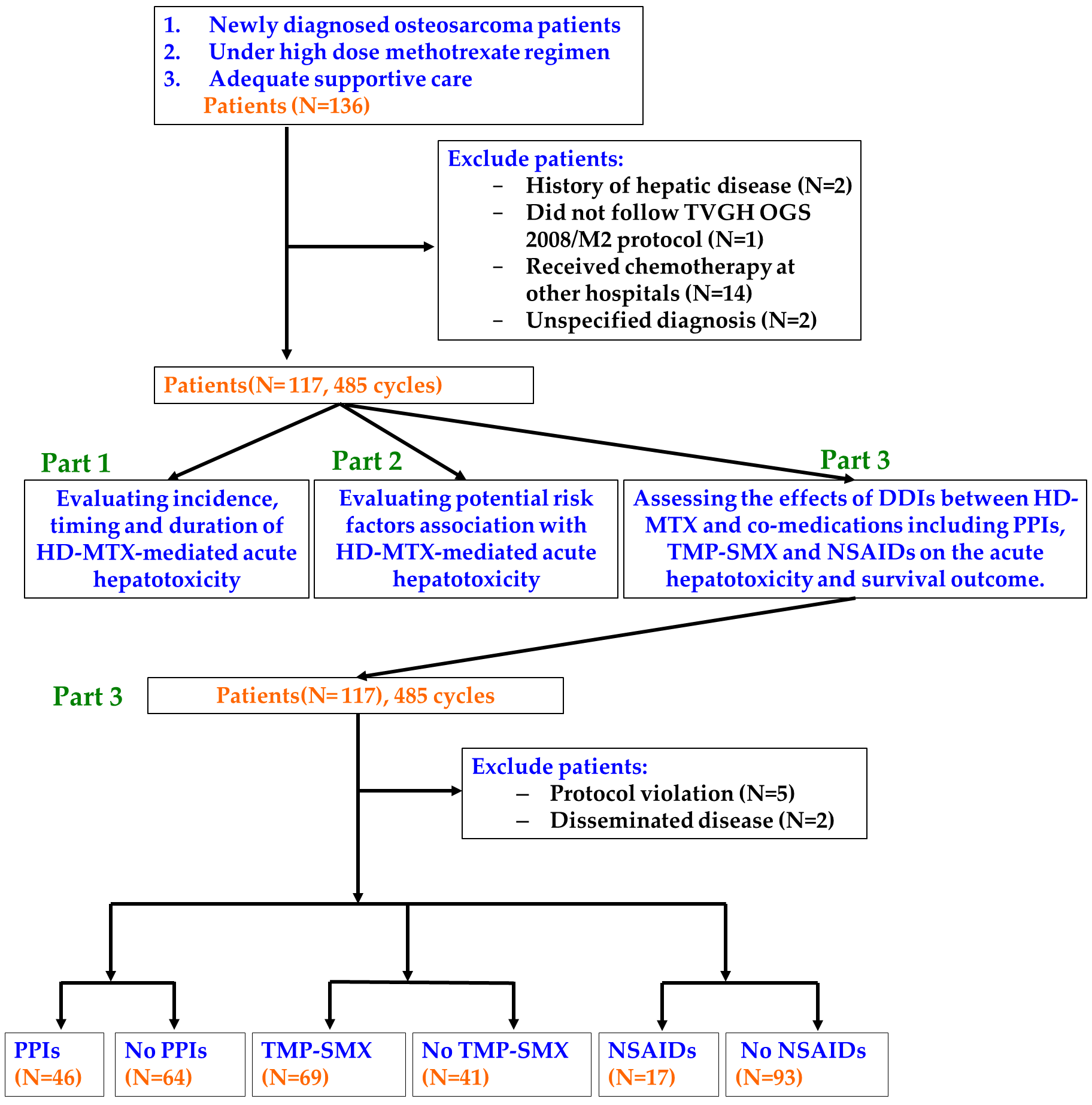
**

**Supplementary Figure 1. Flow chart of data processing.** TVGH, Taipei veteran general hospital; OGS, osteosarcoma; HD-MTX, high-dose methotrexate; PPIs, proton pump inhibitors; TMP-SMX, trimethoprim-sulfamethoxazole; NSAIDs, nonsteroidal anti-inflammatory drugs.


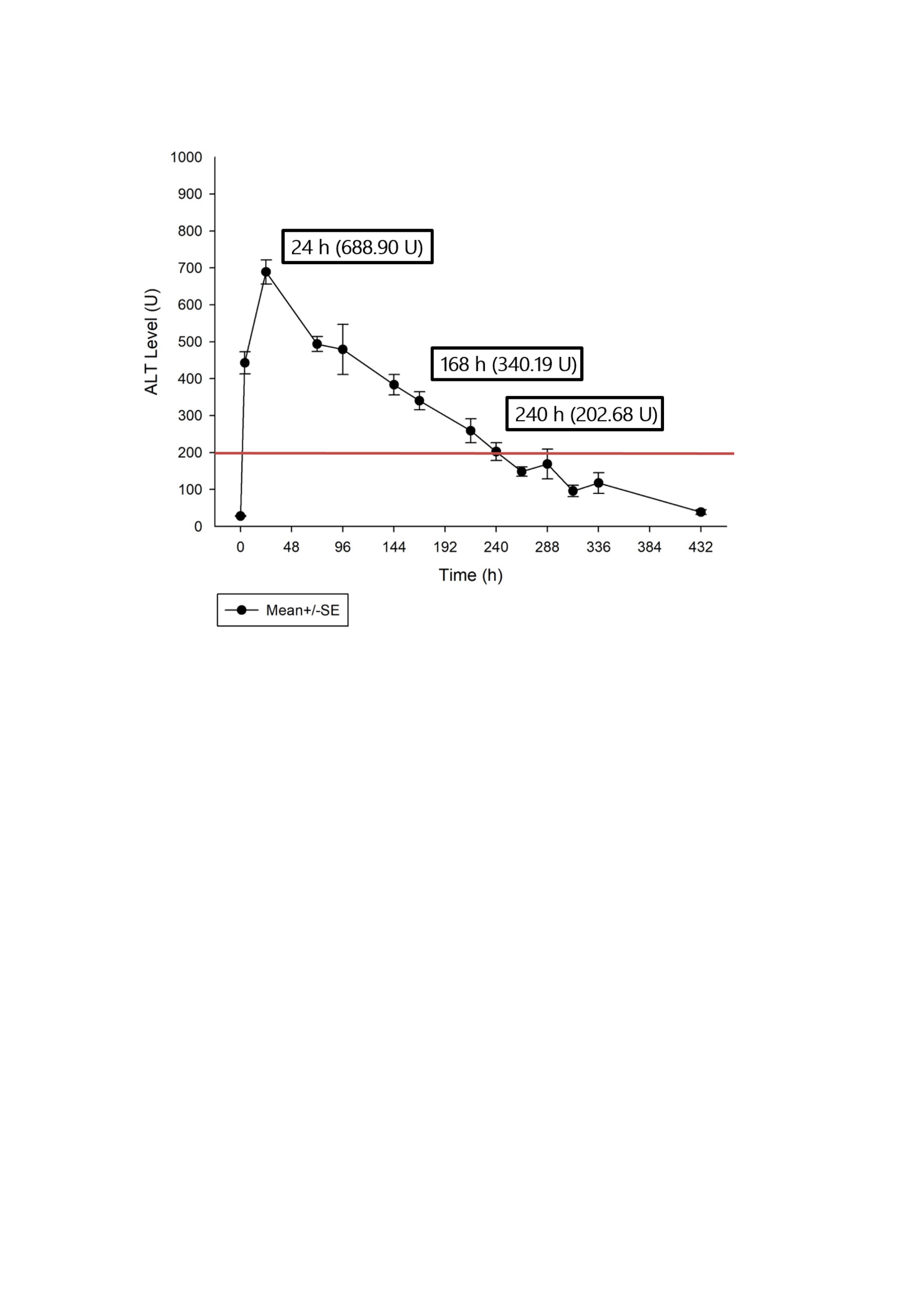


**Supplementary Figure 2. Trend of ALT level after the administration of high-dose methotrexate in osteogenic sarcoma patients with grade ≥ 3 ALT increase.**


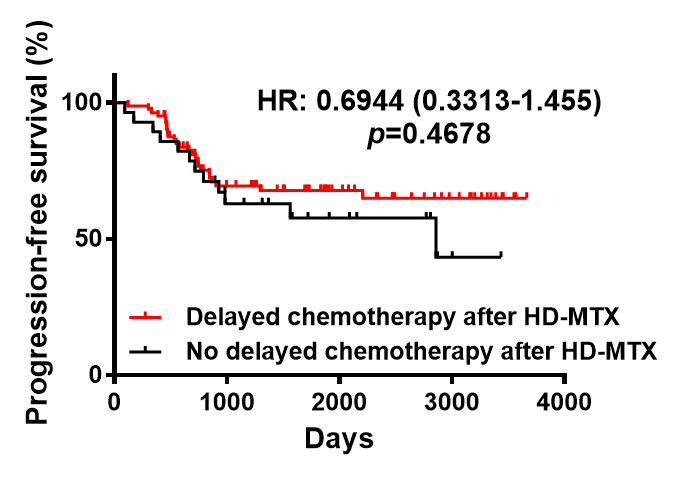


**Supplementary Figure 3. Effects of delayed chemotherapy (>3 days) after HD-MTX on the progression-free survival.**
